# Efficacy and safety of crovalimab versus eculizumab in complement inhibitor-naïve patients with paroxysmal nocturnal hemoglobinuria: Chinese subgroup analysis of the phase 3 COMMODORE 2 study

**DOI:** 10.1007/s00277-026-07129-3

**Published:** 2026-06-13

**Authors:** Hongyan Tong, Zenghua Lin, Chen Yang, Xiaoqin Wang, Jing Sun, Linghui Xia, Jane Shi, Dayu Shi, Zilu Zhang, Anita Appius, Guangsheng He

**Affiliations:** 1 Department of Hematology, First Affiliated Hospital of Zhejiang University School of Medicine, Hangzhou, 310003 China; 2 Department of Hematology, Affiliated Hospital of Nantong University, Nantong, 226001 China; 3 Department of Hematology, Peking Union Medical College, Peking Union Medical College Hospital, Chinese Academy of Medical Sciences, Beijing, 100730 China; 4 Department of Hematology, Huashan Hospital, Fudan University, Shanghai, 200040 China; 5 Department of Hematology, Nanfang Hospital, Southern Medical University, Guangzhou, 510515 China; 6 Department of Hematology, Union Hospital, Tongji Medical College, Huazhong University of Science and Technology, Wuhan, 430022 China; 7 Roche Product Development, Shanghai, 201203 China; 8 F. Hoffmann-La Roche Ltd, Basel, Switzerland; 9 Department of Hematology, Jiangsu Provincial People’s Hospital, First Affiliated Hospital of Nanjing Medical University, Nanjing, 210029 China

**Keywords:** Crovalimab, Eculizumab, Paroxysmal nocturnal hemoglobinuria, China

## Abstract

Paroxysmal nocturnal hemoglobinuria (PNH) is a rare disease with limited treatment options. The COMMODORE 2 study demonstrated that the novel C5 inhibitor crovalimab had comparable safety and non-inferior efficacy to eculizumab. We evaluated the safety and efficacy of crovalimab compared with eculizumab in the Chinese subpopulation of the global COMMODORE 2 study. Adult patients with PNH without previous complement inhibitor therapy were randomized (2:1) to crovalimab or eculizumab for 24 weeks. The co-primary endpoints were the proportions of patients achieving hemolysis control from Weeks 5–25 and those with transfusion avoidance from baseline to Week 25. The secondary endpoints included the proportion of patients with breakthrough hemolysis and hemoglobin stabilization from baseline to Week 25 and the mean change from baseline in FACIT-Fatigue score at Week 25. Safety data were assessed from baseline to the clinical cut-off (16 November 2022). The demographic and baseline characteristics of the 81 patients included (eculizumab: 27; crovalimab: 54) were well-balanced. In the crovalimab and eculizumab arms, respectively, hemolysis control was achieved in 88.3% and 86.8%, transfusion avoidance in 72.2% and 74.1%, hemoglobin stabilization in 72.2% and 70.4%, and breakthrough hemolysis in 5.6% and 11.1%. The FACIT-Fatigue score improvement was greater with crovalimab. Adverse events (81.5% vs. 88.9%) and grade 3–5 adverse events (24.1% vs. 33.3%) were less frequent with crovalimab. Over 24 weeks, 91% of patients achieved complete terminal complement inhibition with crovalimab. Crovalimab demonstrated at least comparable treatment performance and acceptable safety in Chinese patients with PNH.

**Clinical trial registration**

ClinicalTrials.gov identifier NCT04434092 (study registration was first posted on 16th June 2020).

**Supplementary Information:**

The online version contains supplementary material available at https://doi.org/10.1007/s00277-026-07129-3.

## Introduction

Paroxysmal nocturnal hemoglobinuria (PNH) is a rare, acquired stem cell disorder. Data on its prevalence are scarce, but it is estimated to affect 15.9 people per million globally [1]. In Asia, the incidence of PNH is 10 people per million per year [2], but this could be an underestimation due to undiagnosed asymptomatic individuals or comorbidities obscuring diagnosis [3]. In the International PNH Registry, the median age at PNH diagnosis is 42 years [4]. In China, one study reported that among 512 eculizumab-naïve patients with PNH, the median age at onset was 33 years [5].

In PNH, a genetic mutation results in a deficiency in specific proteins responsible for protecting red blood cells and platelets. This deficiency can lead to anemia, hemoglobinuria, and potentially fatal thromboembolic events, which are the leading cause of mortality in patients with PNH [6]. Additionally, PNH often impacts patients’ quality of life and ability to work [7].

Complement inhibitors reduce symptoms and complications in patients with PNH [8, 9]. Before the introduction of eculizumab, PNH was fatal in approximately 35% of patients within 5 years of diagnosis, reaching 50% within 10 years of diagnosis [4, 10]. Eculizumab significantly reduces intravascular hemolysis, stabilizes hemoglobin, reduces the need for red blood cell transfusion, and reduces thromboembolic events and long-term mortality [11]. One study showed that patients with PNH who were continuously treated with eculizumab had a similar life expectancy to age-matched controls [12]. Therefore, eculizumab has several therapeutic benefits in patients with PNH.

Many patients treated with eculizumab require regular blood transfusion because of breakthrough hemolysis (BTH) [9], which may be due to suboptimal C5 inhibition [13], which increases the risk of thrombotic events, leading some patients to require higher doses or more frequent administration of the drug to control BTH [12, 14]. In the global COMMODORE 2 study (NCT04434092) [15], 14.5% of patients treated with eculizumab experienced BTH [16]. Other studies have reported BTH rates ranging from 5.1% to 27% with eculizumab [11, 17–20]. Moreover, eculizumab is extremely costly [21].

Crovalimab is a novel anti-human C5 monoclonal antibody that binds to C5 with high affinity, inhibiting cleavage to C5a and C5b and preventing terminal complement complex C5b-9 (membrane attack complex) generation, membrane disruption, and consequent cell lysis. Compared with eculizumab and ravulizumab, crovalimab binds to a different C5 epitope [22], and it has been shown in ex vivo experiments to block hemolysis in patients with a single missense C5 heterozygous mutation who do not respond to eculizumab or ravulizumab [23]. Moreover, crovalimab has a long half-life (59 days) and reduces free target (C5) accumulation, enabling dosing up to every 4 weeks under the recommended dosing regimen. Crovalimab can be administered subcutaneously, allowing self-administration or assisted administration, either at home or in a healthcare setting [22, 24, 25], increasing patient convenience.

The phase 3 COMMODORE 1 study showed complete terminal complement inhibition with crovalimab [26], which is important because suboptimal terminal complement inhibition accounts for most cases of BTH with eculizumab [13]. Moreover, the phase 3 COMMODORE 3 study evaluated the safety and efficacy of crovalimab in complement inhibitor-naïve Chinese patients with PNH [27]. All treated patients achieved hemolysis control and transfusion avoidance. Crovalimab was efficacious and well tolerated with subcutaneous dosing every 4 weeks [27]. The phase 3 COMMODORE 2 study [15,16] demonstrated that crovalimab was non-inferior to eculizumab for hemolysis control, transfusion avoidance, BTH, and hemoglobin stabilization. A clinically meaningful improvement in Functional Assessment of Chronic Illness Therapy – Fatigue (FACIT-Fatigue) score [28] was also observed. Moreover, complete terminal complement inhibition was generally maintained with crovalimab. Most patients reported a preference for crovalimab after switching from eculizumab.

The COMMODORE studies highlight the favorable benefit–risk profile of crovalimab in PNH patients [16]. However, the safety and efficacy of crovalimab compared with eculizumab are yet to be evaluated in complement inhibitor-naïve Chinese patients with PNH. The present study is a subgroup analysis of COMMODORE 2, which was conducted to evaluate the safety and efficacy of crovalimab compared with eculizumab in Chinese complement inhibitor-naïve patients with PNH.

## Methods

### Study design and patients

**Fig. 1:**
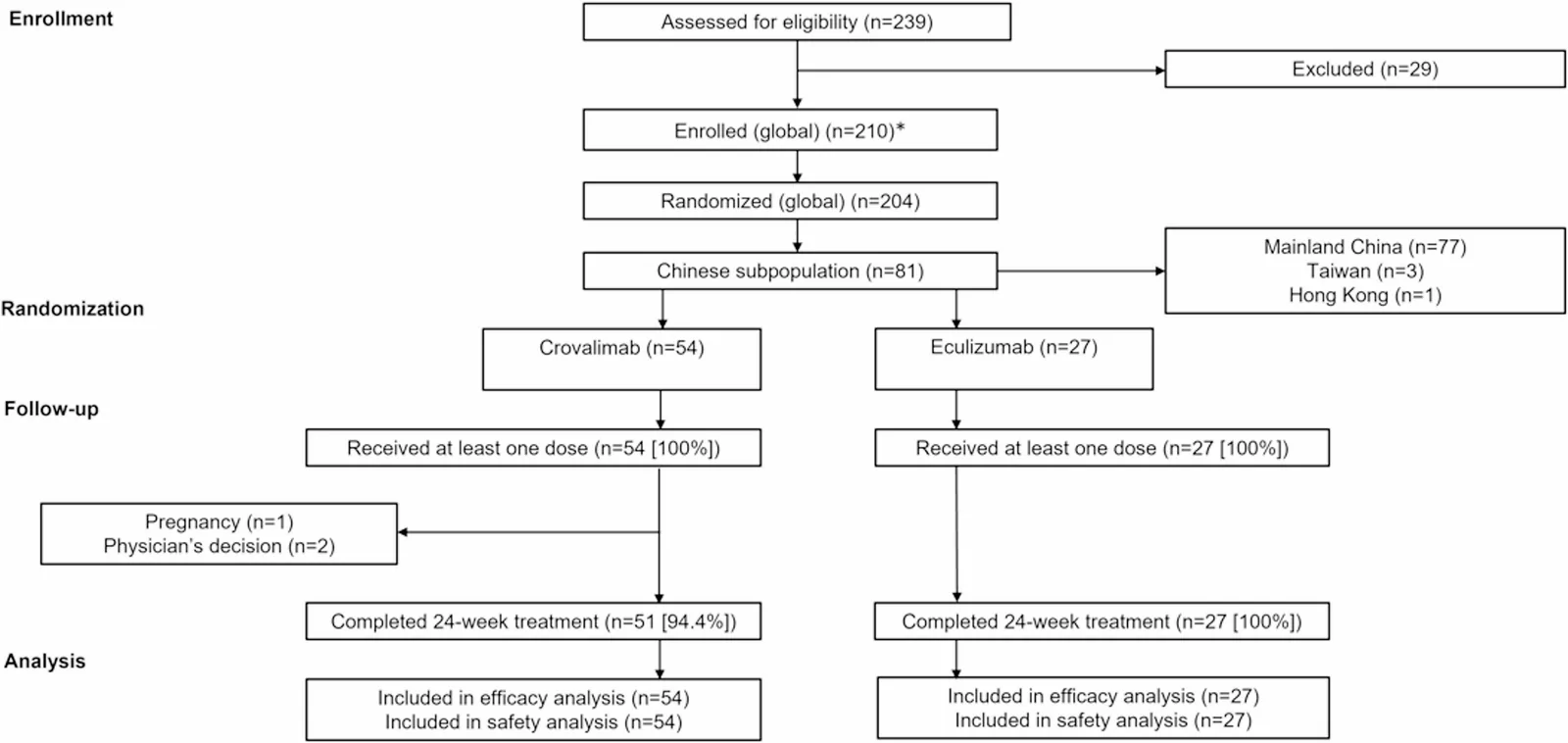
Flow diagram of patient enrolment, randomization, follow-up, and analysis. *Six pediatric patients entered the non-randomized arm and were treated with crovalimab

The full details of the COMMODORE 2 study are reported elsewhere [15,16]. In this subanalysis, the data of Chinese complement inhibitor-naïve patients with PNH aged ≥ 18 years who were randomized 2:1 to crovalimab or eculizumab were analyzed. The full patient selection criteria have been described previously [15,16]. For this Chinese subpopulation analysis, the key inclusion and exclusion criteria are provided in the Online Resource - Methods. The patient flow is shown in Fig. 1. The screening period lasted for up to 28 days. The primary treatment period was 24 weeks, and the primary analysis was conducted when all randomized patients had completed this period or discontinued treatment, whichever came first. After the 24-week treatment period, patients randomized to eculizumab had the option to switch to crovalimab in the extension period, which was no more than 5 years. The first Chinese patient was enrolled on 28 December 2020, and the clinical data cut-off was 16 November 2022. The study was not blinded to the patients or the investigators. To maximize the study’s integrity, the sponsor had no access to the aggregated data by treatment arm until the primary analysis.

### Ethics

The COMMODORE 2 study complied with the ethical standards of the responsible committee on human experimentation and with the Declaration of Helsinki of 1975, revised in 2013. Approval was obtained from the institutional review board of each participating site. Written informed consent was obtained from all patients. The COMMODORE 2 study is registered at ClinicalTrials.gov (NCT04434092) [14].

### Dosing and administration

The drug administration schedules are summarized in Online Resource Fig. 1. Patients randomized to crovalimab received a weight-based tiered dosing regimen. Patients randomized to eculizumab received a 600-mg loading dose of eculizumab intravenously on Days 1, 8, 15, and 22, followed by intravenous maintenance doses of 900 mg on Day 29 and every 2 weeks thereafter.

### Efficacy assessments

The primary and secondary efficacy analyses of crovalimab versus eculizumab included all randomized patients who received at least one dose and who had ≥ 1 lactate dehydrogenase (LDH) measurement after the first infusion on Week 1, Day 1. The co-primary efficacy endpoints were hemolysis control (mean proportion of patients with LDH ≤ 1.5 × upper limit of normal [ULN] from Week 5 through Week 25) and transfusion avoidance (proportion of patients who did not require transfusion from baseline through Week 25). The secondary efficacy endpoints were the proportions of patients with BTH [15,16], hemoglobin stabilization, and change in FACIT-Fatigue score [29] from baseline to Week 25. BTH was defined as at least one new or worsening symptom or sign of intravascular hemolysis [16] in the presence of elevated LDH ≥ 2 × ULN after prior reduction of LDH to ≤ 1.5 × ULN on treatment [16]. Actual observed BTH refers to breakthrough hemolysis events directly observed during the study, whereas a conservative estimation reflects an analysis approach that accounts for intercurrent events in the evaluation of BTH rates. Hemoglobin stabilization was defined as avoidance of a decrease of ≥ 2 g/L in hemoglobin from baseline in the absence of transfusion [16]. The FACIT-Fatigue score is a 13-item patient-reported outcome instrument designed to assess fatigue-related symptoms and impact on daily functioning, with the total score ranging from 0 to 52 [16, 29].

### Safety assessments

Safety assessments were performed on all randomized patients who received ≥ 1 dose of study drug, with patients grouped according to the treatment received. Safety assessments included the incidence and severity of adverse events (AEs), with severity determined according to the National Cancer Institute’s Common Terminology Criteria for Adverse Events, version 5 [30]. The incidence of serious AEs, AEs of special interest [15, 16], and AEs leading to study drug discontinuation were assessed. Other safety assessment details are provided in the Online Resource - Methods.

### Pharmacokinetics, pharmacodynamics, and immunogenicity

The pharmacokinetic, pharmacodynamic, and immunogenicity assessments are described in the Online Resource - Methods.

### Statistical analysis

The demographic and baseline characteristics of the patients are summarized as the mean ± standard deviation or standard error and/or median (range) for continuous variables, and as number (proportion) for categorical variables. The 95% confidence intervals (CIs) for the proportion of patients with hemolysis control, transfusion avoidance, BTH, and hemoglobin stabilization in each arm were calculated using Wilson’s method with continuity correction [31]. The 95% CIs for the difference in the proportion of patients with hemolysis control, transfusion avoidance, BTH, and hemoglobin stabilization between the arms were calculated using the Stratified Newcombe CI method [31]. For the change from baseline in the FACIT-Fatigue score, a score difference of ≥ 5 points indicated a clinically meaningful difference. When estimating odds ratios (OR) for hemolysis control, an AR (1) covariance matrix structure was assumed to account for intra-individual correlation of repeated central LDH measurements across visits. Non-inferiority of crovalimab to eculizumab in the efficacy endpoints was not officially tested in the present Chinese subpopulation analysis; the results are descriptive only.

## Results

### Disposition and demographic/baseline characteristics

In the global COMMODORE 2 study, 204 patients were randomized. Of these, 81 (39.7%) were Chinese and were included in the present analysis (crovalimab, *n* = 54; eculizumab, *n* = 27). There were 77 patients (95.1%) from mainland China, 1 (1.2%) from Hong Kong, and 3 (3.7%) from Taiwan (Fig. 1). All patients in the Chinese subpopulation received ≥ 1 dose of study treatment (100%). Fifty-one of 54 patients (94.4%) randomized to crovalimab and 27 of 27 (100%) randomized to eculizumab completed the 24-week treatment period. Three patients discontinued crovalimab within the 24-week treatment period, but no patient discontinued treatment because of death. All patients (27/27 [100%]) originally randomized to eculizumab switched to crovalimab in the extension period.

The demographic and baseline characteristics were generally balanced across the two arms (Table 1). The median age was 35 (18–71) years in the crovalimab arm and 36 (17–74) years in the eculizumab arm. Although age ≥ 18 years was one of the selection criteria, one patient aged < 18 years was included in the eculizumab arm. In the crovalimab arm, 25/54 patients (46.3%) were male versus 14/27 (51.9%) in the eculizumab arm. The median weight at baseline was 61.5 (42.0–140.3) kg and 66.0 (47.0–122.0) kg in the crovalimab and eculizumab groups, respectively. Most patients (96.3%) weighed between ≥ 40 kg and < 100 kg at baseline (eculizumab, 26/27; crovalimab, 52/54). The median time from PNH diagnosis to enrollment was comparable between the arms (crovalimab: 4.6 [0.1–31.9] years; eculizumab: 4.3 [0.1–20.0] years). The mean LDH concentration at baseline (× ULN) was 7.93 ± 3.11 in the crovalimab arm and 7.89 ± 2.40 in the eculizumab arm. The baseline median hemoglobin concentration was also balanced between the crovalimab (81.0 [63.0–131.0] g/L) and eculizumab (82.0 [58.0–101.0] g/L) arms.

**Table 1:** Patients’ demographic and baseline characteristics

		Eculizumab (*n* = 27)	Crovalimab (*n* = 54)
Demographic characteristics			
Age (years), median (range)		36 (17–74)	35 (18–71)
Age (years, categories), n (%)	< 18 ≥ 18 to < 64 ≥ 64	1 (3.7) 24 (88.9) 2 (7.4)	0 51 (94.4) 3 (5.6)
Sex, n (%)	Male	14 (51.9)	25 (46.3)
Race, n (%)	Asian	27 (100)	54 (100)
Weight (kg), median (range)		66.0 (47.0–122.0)	61.5 (42.0–140.3)
Weight (kg, categories), n (%)	≥ 40 to < 100 ≥ 100	26 (96.3) 1 (3.7)	52 (96.3) 2 (3.7)
Baseline characteristics			
Hemoglobin (g/L), median (range)		82.0 (58.0–101.0)	81.0 (63.0–131.0)
Local LDH value at baseline (× ULN), mean (SD)		7.89 (2.40)	7.93 (3.11)
Local LDH value at baseline (× ULN, categories), n (%)	≤ 2 > 2 to ≤ 4 > 4	0 2 (7.4) 25 (92.6)	0 5 (9.4) 48 (90.6)
pRBC units transfused within 12 months before screening (units), mean (SD)		6.61 (6.45)	8.51 (10.60)
pRBC units transfused within 12 months before screening (categories), n (%)	0 < 4 ≥ 4 to ≤ 14 > 14	7 (25.9) 4 (14.8) 11 (40.7) 5 (18.5)	6 (11.1) 16 (29.6) 21 (38.9) 11 (20.4)
History of PNH-relevant conditions before enrollment/first dose administration, n (%)	Aplastic anemia Myelodysplastic syndrome Renal impairment (eGFR < 60 mL/min/1.73 m^2^) Major vascular events	14 (51.9) 2 (7.4) 4 (14.8) 3 (11.1)	18 (33.3) 0 5 (9.3) 10 (18.5)

*eGFR* estimated glomerular filtration rate, *LDH* lactate dehydrogenase, *pRBC* packed red blood cell, *PNH* paroxysmal nocturnal hemoglobinuria, *SD* standard deviation, *ULN* upper limit of normal

### Treatment exposure

The median treatment duration was similar between the crovalimab (20.14 [3.4–22.1] weeks) and eculizumab (22.14 [21.9–23.1] weeks) arms. The median number of doses administered was 10 (4–12) in the crovalimab arm and 14 (13–14) in the eculizumab arm. Between Weeks 9 and 25, most subcutaneous injections were administered by healthcare professionals; however, an increasing proportion (26.9%–43.1%) of patients self-administered or had crovalimab administered by a caregiver up to Week 25 and thereafter. No patients had a medication error that led to dose modification. Only one patient received unscheduled intravenous crovalimab as rescue therapy during the primary treatment period.

### Efficacy assessments

The results of the co-primary and secondary efficacy assessments are shown in Table 2.

**Table 2:** Primary and secondary efficacy outcome assessment results

		Eculizumab (*n* = 27)	Crovalimab (*n* = 54)
Co-primary endpoint	Mean proportion of patients achieving hemolysis control (central LDH ≤ 1.5 × ULN), % (95% CI) Odds ratio (95% CI)	86.8 (74.35, 93.74) 1.14 (0.43, 3.06)	88.3 (80.94, 93.04)
Co-primary endpoint	Proportion of patients with transfusion avoidance from baseline through Week 25, % (95% CI) Weighted difference in proportion, % (95% CI)	74.1 (53.41, 88.13) −4.8 (− 23.88, 17.20)	72.2 (58.14, 83.14)
Secondary endpoint	Proportion of patients with BTH from baseline through Week 25, % (95% CI) Weighted difference in proportion, % (95% CI)	11.1 (2.91, 30.30) 1.60 (− 17.62, 16.02)	11.1 (4.60, 23.31)
Secondary endpoint	Proportion of patients with stabilized hemoglobin from baseline through Week 25, % (95% CI) Weighted difference in proportion, % (95% CI)	70.4 (49.66, 85.50) −0.9 (− 20.42, 21.12)	72.2 (58.14, 83.14)
Secondary endpoint	Adjusted mean change from baseline to Week 25 in FACIT-Fatigue score (95% CI) Difference in mean change from baseline scores (95% CI)	5.64 (2.70, 8.58) 0.86 (− 2.18, 3.89)	6.50 (4.26, 8.74)

*BTH* breakthrough hemolysis, *CI* confidence interval, *FACIT-Fatigue* Functional Assessment of Chronic Illness Therapy – Fatigue, *LDH* lactate dehydrogenase, *ULN* upper limit of normal

### Co-primary efficacy endpoints

**Fig. 2:**
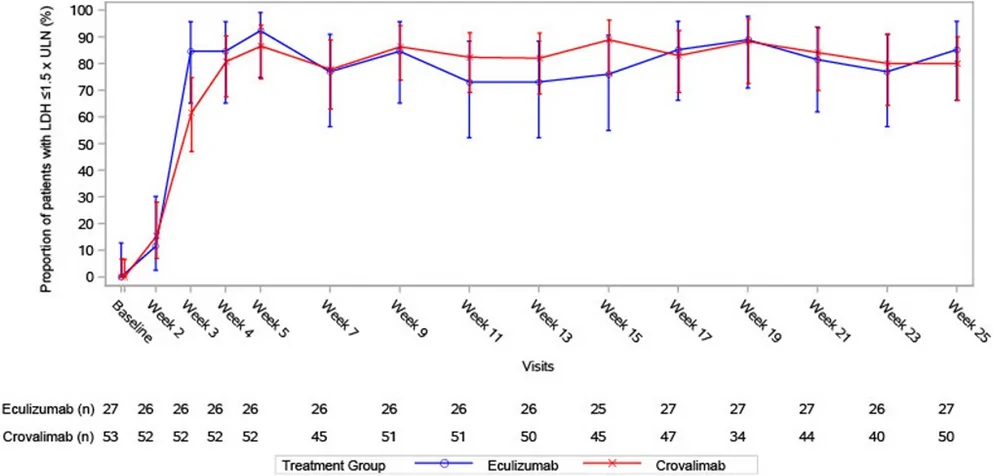
Proportion of patients treated with crovalimab or eculizumab who achieved LDH ≤ 1.5 × ULN from baseline through week 25. LDH: lactate dehydrogenase; ULN: upper limit of normal

**Fig. 3:**
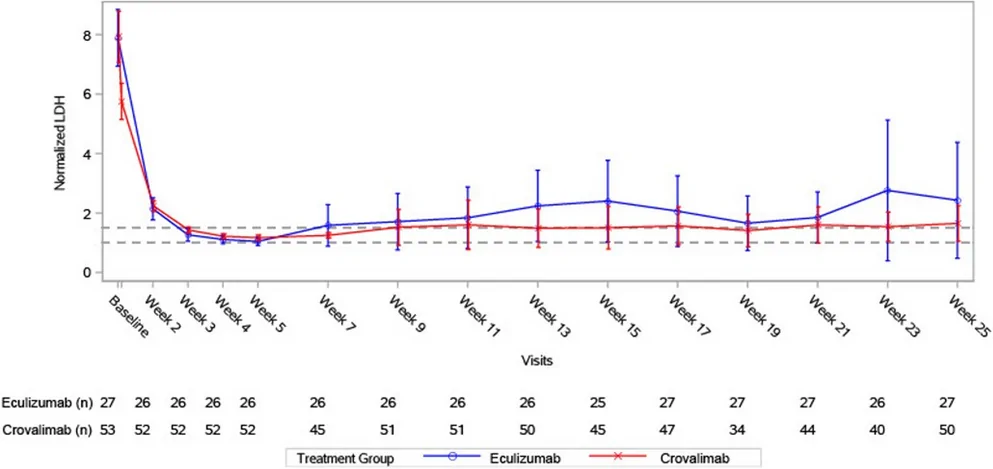
Mean normalized LDH by visit during the primary treatment period in patients treated with eculizumab or crovalimab. LDH: lactate dehydrogenase

Overall, the mean proportion of patients achieving hemolysis control was 88.3% (95% CI 80.94%, 93.04%) in the crovalimab arm and 86.8% (95% CI 74.35%, 93.74%) in the eculizumab arm at Week 25. The OR for hemolysis control was 1.14 (95% CI 0.43, 3.06), favoring crovalimab. The mean proportion of patients with LDH ≤ 1.5 × ULN with hemolysis control steadily increased from baseline to Week 5 (as expected), stabilized, and was maintained through Week 25 in both arms (Fig. 2). LDH ≤ 1.5 × ULN was reached at Week 3 in both arms (Fig. 3). In the crovalimab arm, mean normalized LDH was maintained close to 21.5 × ULN through Week 25. However, in the eculizumab arm, mean normalized LDH was consistently > 1.5 × ULN from Week 7 through Week 25.

The proportion of patients with transfusion avoidance from baseline through Week 25 was 72.2% (39/54; 95% CI 58.14%, 83.14%) in the crovalimab arm and 74.1% (20/27; 95% CI 53.41%, 88.13%) in the eculizumab arm (weighted difference: −4.8%; 95% CI − 23.88%, 17.20%).

### Secondary efficacy endpoints

Three patients (5.6%) in the crovalimab arm were conservatively considered to experience a BTH event after discontinuing treatment during the primary treatment period. The proportion of patients with BTH from baseline through Week 25 according to conservative estimation was 11.1% (6/54; 95% CI 4.60%, 23.31%) in the crovalimab arm and 11.1% (3/27; 95% CI 2.91%, 30.30%) in the eculizumab arm (weighted difference: 1.60%; 95% CI − 17.62%, 16.02%). The proportion of patients with actual observed BTH was 5.6% (3/54) in the crovalimab arm and 11.1% (3/27) in the eculizumab arm.

The proportion of patients with hemoglobin stabilization from baseline through Week 25 was 72.2% (39/54; 95% CI 58.14%, 83.14%) in the crovalimab arm and 70.4% (19/27; 95% CI 49.66%, 85.50%) in the eculizumab arm (weighted difference: −0.9%; 95% CI − 20.42%, 21.12%).

The baseline mean ± standard error FACIT-Fatigue score was 36.24 ± 1.38 in the crovalimab arm and 35.08 ± 2.17 in the eculizumab arm. The adjusted mean change from baseline to Week 25 in the FACIT-Fatigue score was 6.50 (95% CI 4.26, 8.74) in the crovalimab arm and 5.64 (95% CI 2.70, 8.58) in the eculizumab arm, with a difference in the adjusted mean change from baseline of 0.86 ± 1.52 (95% CI − 2.18, 3.89). The adjusted mean change from baseline to Week 25 in the FACIT-Fatigue score exceeded the threshold for clinically meaningful improvement (≥ 5 points).

Overall, the descriptive results for hemolysis control, transfusion avoidance, BTH, hemoglobin stabilization, and FACIT-Fatigue score were similar between crovalimab and eculizumab, and crovalimab had a comparable treatment effect to eculizumab for these efficacy endpoints.

### Safety assessments

The proportion of patients with at least one AE was higher with eculizumab (24/27 [88.9%] vs. 44/54 [81.5%]). Treatment-related AEs were more frequent with eculizumab (14/27 [51.9%] vs. 22/54 [40.7%]).

Grade 3–5 AEs were more frequent with eculizumab (9/27 [33.3%] vs. 13/54 [24.1%]) (Table 3). Except for decreased neutrophil count (crovalimab: 6/54 [11.1%]; eculizumab: 5/27 [18.5%]), which was lower with crovalimab, and decreased white blood cell count (crovalimab: 3/54 [5.6%]; eculizumab: 1/27 [3.7%]), all other grade 3–5 AEs occurred in a single patient. Death resulting from an AE occurred in one patient (1.9%) treated with crovalimab. The event was respiratory tract hemorrhage that was considered unrelated to crovalimab. The patient discontinued treatment at the physician’s discretion on Day 24. Death occurred on Day 151, after the patient had reported AEs of lung neoplasm and COVID-19 on Days 113 and 140, respectively. Therefore, although one death occurred in the crovalimab group that was unrelated to the drug, the Grade 3/4 event burden was higher in the eculizumab group.

**Table 3:** Summary of AEs, serious AEs, and grade 3–5 AEs in patients treated with crovalimab and eculizumab

	Eculizumab (*n* = 27)	Crovalimab (*n* = 54)
Patients with at least one AE	24 (88.9)	44 (81.5)
Total number of AEs, n	118	212
Patients with serious AEs	3 (11.1)	6 (11.1)
Patients with grade 3–5 AEs	9 (33.3)	13 (24.1)
Patients with treatment-related AEs	14 (51.9)	22 (40.7)
Patients with treatment-related serious AEs	1 (3.7)	3 (5.6)
Patients with AEs leading to dose modification/interruption	1 (3.7)	1 (1.9)
Patients with treatment-related AEs leading to dose modification/interruption	0 (0)	1 (1.9)
Patients with AEs leading to treatment withdrawal	0 (0)	0 (0)
Patients with treatment-related AEs leading to treatment withdrawal	0 (0)	0 (0)
Patients with AEs leading to study withdrawal	0	0
Total number of deaths	0	1 (1.9)
Patients with at least one AE with a fatal outcome	0	1 (1.9)

*AE* adverse event

Data are n (%) unless otherwise stated

The total number of patients with at least one serious AE was 6/54 (11.1%) in the crovalimab arm and 3/27 (11.1%) in the eculizumab arm. All serious AEs occurred in a single patient. No patients had protocol-defined AEs of special interest (drug-induced liver injury, transmission of an infectious agent, or type III hypersensitivity reaction).

The rates of commonly reported AEs were generally comparable between arms, except for upper respiratory tract infection, which was > 20% higher with eculizumab than with crovalimab (29.6% vs. 9.3%) (Table 4).

**Table 4:** Adverse events that occurred in ≥ 10% of patients

Preferred term	Eculizumab (*n* = 27)	Crovalimab (*n* = 54)
White blood cell count decreased, n (%)	7 (25.9)	16 (29.6)
Neutrophil count decreased, n (%)	7 (25.9)	16 (29.6)
Hypokalemia, n (%)	8 (29.6)	13 (24.1)
Hyperuricemia, n (%)	6 (22.2)	11 (20.4)
Hypocalcemia, n (%)	6 (22.2)	8 (14.8)
Upper respiratory tract infection, n (%)	8 (29.6)	5 (9.3)
Infusion-related reaction, n (%)	4 (14.8)	4 (7.4)
Pyrexia, n (%)	3 (11.1)	2 (3.7)

### Pharmacokinetics

In both treatment-naïve patients (patients originally randomized to crovalimab) and patients who switched from eculizumab to crovalimab in the extension period, crovalimab exposure plateaued by Week 13, indicating steady-state exposure, and remained stable up to Week 25. The crovalimab concentration was above the threshold for complete terminal complement inhibition (~ 100 g/mL) in 90.7% of the patients (49/54) originally randomized to crovalimab and in 100% (26/26) who switched to crovalimab. In both arms, complement activity levels (CH50) reduced from a mean baseline of ~ 50 U/mL to very low levels close to or below the quantification limit (< 10 U/mL) from Week 2.

### Pharmacodynamics

In the crovalimab arm, free C5 was reduced to low levels (< 0.0001 g/L) from Week 2 of treatment. Low free C5 was generally sustained throughout treatment, demonstrating complete terminal complement inhibition. In the crovalimab arm, the mean total C5 plateaued at Week 4 at around 0.2 g/L compared with 0.3 g/L in the eculizumab arm.

### Immunogenicity

At baseline, 3/54 patients (5.6%) in the crovalimab arm were positive for anti-drug antibodies (ADAs). Treatment-emergent ADAs with crovalimab were detected in 18/54 patients (33.3%). Of these, one patient (1.9%) had treatment-enhanced ADAs and 17 (31.5%) had treatment-induced ADAs. Of the 18 patients with treatment-emergent ADAs, neutralizing antibody results were available for 16, two of whom were positive for neutralizing ADA, which led to complete loss of exposure. Overall, ADA-positive patients had slightly lower serum crovalimab concentrations than ADA-negative patients. However, at steady-state (Week 13), the mean crovalimab concentration in ADA-positive patients was approximately 200 g/mL and remained above the threshold (~ 100 g/mL) for terminal complement inhibition. Moreover, the profiles of free C5, CH50, and LDH over time showed no relevant differences in the majority of ADA-positive and ADA-negative patients.

### Patient treatment preference

Of the 18/27 patients who reported their treatment preference after switching to crovalimab in the extension period, one (5.6%) preferred eculizumab, 15 (83.3%) preferred crovalimab, and two (11.1%) had no preference.

## Discussion

The results of this descriptive analysis of data from the Chinese subpopulation of the global COMMODORE 2 study were generally consistent with the results of the full global study [16] in the sense that, in both studies, crovalimab had comparable efficacy to eculizumab for hemolysis control, transfusion avoidance, BTH, hemoglobin stabilization, and FACIT-Fatigue score. The global study showed that crovalimab was non-inferior to eculizumab for these efficacy endpoints in complement inhibitor-naïve patients with PNH, suggesting that this trend may also be present in the Chinese subpopulation, although non-inferiority was not officially tested.

The characteristics of patients included in the COMMODORE 2 study, and thus the present subpopulation, are consistent with those of a treatment-naïve PNH population [4]. LDH was ≥ 2 × ULN at baseline in all patients, with > 80% having LDH ≥ 4 × ULN. The baseline characteristics of the Chinese subpopulation were generally consistent with the global population [16], except that a higher proportion of the Chinese patients had a baseline local LDH value > 4 × ULN and had undergone packed red blood cell transfusion ≥ 4 units within 12 months before screening, indicating uncontrolled hemolysis and the need for pretreatment transfusion. The comparability of the baseline characteristics between the global population and the Chinese subpopulation indicates the suitability of the population studied.

Eculizumab was chosen as the comparator for crovalimab, as ravulizumab had not yet been approved or made available for PNH treatment in China. While the safety and efficacy of crovalimab have been compared with eculizumab in PNH patients previously treated with complement inhibitors (COMMODORE 1) [26], and the safety and efficacy of crovalimab alone have been evaluated in Chinese complement inhibitor-naïve PNH patients (COMMODORE 3), COMMODORE 2 evaluated the safety and efficacy of crovalimab compared with eculizumab in the global population of complement inhibitor-naïve patients with PNH [16]. However, before the present Chinese subpopulation analysis, the safety and efficacy of crovalimab had not been compared with eculizumab specifically in Chinese PNH patients. Therefore, this subgroup analysis of COMMODORE 2 provides additional insights regarding the safety and efficacy of crovalimab compared with eculizumab in complement inhibitor-naïve Chinese patients with PNH.

The eculizumab arm demonstrated the known treatment effects of eculizumab [17, 18, 32]. In terms of the co-primary efficacy endpoints, the proportions of patients with hemolysis control (OR 1.14; 95% CI 0.43, 3.06) and transfusion avoidance (weighted difference in proportion − 4.8%; 95% CI − 23.88%, 17.20%) were similar between eculizumab and crovalimab. In terms of the similarity in the results between eculizumab and crovalimab, these findings are consistent with the global COMMODORE 2 population (hemolysis control: OR 1.02; 95% CI 0.57, 1.82; transfusion avoidance: weighted difference in proportion − 2.8%; 95% CI − 15.67%, 11.14%) [15]. In the global study, the non-inferiority of crovalimab was concluded for hemolysis control if the 95% CI lower limit was greater than the pre-defined non-inferiority margin of 0.2, and for transfusion avoidance if the 95% CI lower limit was higher than the pre-defined non-inferiority margin of − 20%, both of which were met. Although non-inferiority was not officially tested in the present study, the results showed a similar trend to what was observed in the global study in terms of the comparability of the efficacy endpoints between the crovalimab and eculizumab arms. Therefore, future non-inferiority trials in the Chinese subpopulation are expected to produce similar findings to the global study with regards to crovalimab.

Although the efficacy endpoints were comparable between eculizumab and crovalimab in both the global population and the Chinese subpopulation, numerically higher proportions of patients in the Chinese subpopulation than in the global population achieved hemolysis control (Chinese vs. global: crovalimab, 88.3% vs. 79.3%; eculizumab, 86.8% vs. 79.0%) and transfusion avoidance (Chinese vs. global: crovalimab, 72.2% vs. 65.7%; eculizumab, 74.1% vs. 68.1%). These findings indicate numerically higher rates of hemolysis control and transfusion avoidance in the Chinese subpopulation than in the global population [16].

Also of note is that the Chinese subpopulation had lower rates of BTH than the global population with both crovalimab (5.6% vs. 10.4%, respectively) and eculizumab (11.1% vs. 14.5%, respectively). In the present study, the rate of BTH was 5.6% with crovalimab and 11.1% with eculizumab, favoring crovalimab. The COMPOSER trial reported a BTH value per year of crovalimab treatment of 0.13, which compares favorably to the BTH event rates of other treatments [17, 18]. The COMMODORE 3 study also reported a low BTH rate with crovalimab [27]. This suggests that crovalimab has a lower BTH rate than eculizumab in both global and Chinese populations. BTH with eculizumab often occurs because of suboptimal C5 inhibition [17]. Crovalimab has a long half-life (59 days) and reduces free target (C5) accumulation. Moreover, crovalimab binds to a different C5 epitope to eculizumab and ravulizumab [22] and has been shown to block hemolysis in patients who do not respond to either eculizumab or ravulizumab [23]. Furthermore, crovalimab uses a weight-based, subcutaneous dosing schedule that ensures consistent and complete C5 inhibition. This minimizes the risk of C5 inhibition falling below therapeutic levels and thus reducing the risk of BTH. Therefore, crovalimab may be a useful alternative to eculizumab.

For the secondary efficacy endpoint of hemoglobin stabilization, crovalimab showed a comparable treatment effect to eculizumab in the Chinese subpopulation (weighted difference in proportion − 0.9%; 95% CI − 20.42%, 21.12%). These findings are similar to those of the global COMMODORE 2 study (weighted difference in proportion 2.2%; 95% CI − 11.37%, 16.31%), in which the non-inferiority of crovalimab was concluded if the lower limit of the 95% CI for the difference in the proportion of patients with hemoglobin stabilization was greater than the pre-defined non-inferiority margin of − 20% [16]. Similar to the other endpoints discussed, the proportion of patients achieving hemoglobin stabilization in both arms was higher in the Chinese subpopulation than in the global population.

The TRIUMPH and SHEPARD trials showed that eculizumab improves FACIT-Fatigue scores in patients not previously treated with complement inhibitors [9, 32]. The global COMMODORE 2 study also showed that both eculizumab and crovalimab led to a clinically meaningful improvement in fatigue according to the FACIT-Fatigue score at Week 25 [16]. In this Chinese subpopulation analysis of COMMODORE 2, the adjusted mean change from baseline to Week 25 in both the crovalimab and eculizumab arms exceeded the threshold for clinically meaningful improvement (≥ 5 points). The same was observed in the global population of the COMMODORE 2 study [16].

Of the patients who rated their treatment preference after switching from eculizumab to crovalimab, 83.3% preferred crovalimab, corroborating the findings of the global population (84.2%) [16]. One possible reason may be the improvement in FACIT-Fatigue score, which equates to an improvement in fatigue during activities of daily living. As quality of life is important in patients with life-threatening and/or chronic diseases, the greater improvement in fatigue may be one factor contributing to the preference of patients for crovalimab over eculizumab. Another reason for this preference may be that eculizumab requires hospital visits for frequent (biweekly) intravenous dosing, which is time-consuming and inconvenient for patients. Unfortunately, this cannot be resolved by increasing the dosing interval, with a previous study showing that increasing the dosing interval to ≥ 17 days may increase the risk of BTH [19]. Crovalimab can be administered subcutaneously every 4 weeks, eliminating the need for frequent intravenous dosing. Studies have shown that most patients prefer this method as it allows for home administration and offers convenience [33, 34]. This patient preference is important for adherence and quality of life.

Regarding safety, crovalimab was generally well tolerated. Overall, the safety profile of crovalimab was comparable with that of eculizumab. The safety profile was also generally consistent between ADA-positive and ADA-negative patients. The safety results in the Chinese subpopulation were generally consistent with those of the global population of COMMODORE 2 [16]. Specifically, the proportion of patients with AEs, treatment-related AEs, and grade 3–5 AEs was higher with eculizumab than crovalimab in both the Chinese subpopulation and the global population. However, overall, the rates of related AEs and grade 3–5 AEs were higher in the Chinese subpopulation than in the global population in both arms. The overall safety profile of crovalimab was consistent with COMMODORE 1 [26], COMMODORE 3 [27], and COMPOSER [24], indicating the reliability of the results.

### Strengths and limitations

This study represents one of the largest analyses of Chinese complement inhibitor-naïve patients with PNH, addressing a critical gap in data for this demographic. Crovalimab demonstrated comparable efficacy to eculizumab while offering the added benefit of subcutaneous administration with longer dosing intervals, improving patient convenience and reducing the need for frequent hospital visits. However, this subpopulation analysis has some limitations. First, it had a limited sample size; therefore, the findings should be validated in a larger study in China in the future. Second, blinding was not used. Third, the results of the efficacy analyses were not compared against a non-inferiority margin in the Chinese subpopulation, so non-inferiority could not be concluded; rather, the results are descriptive. We acknowledge the exploratory nature of the between-arm comparisons and the statistical limitations of a subgroup analysis of this size. Therefore, future studies should be conducted to formally evaluate whether crovalimab is non-inferior to eculizumab for the treatment of Chinese patients with PNH.

## Conclusion

This exploratory analysis of the Chinese subpopulation based on data from the global COMMODORE 2 study demonstrated at least comparable treatment performance between crovalimab and eculizumab in hemolysis control, transfusion avoidance, BTH, hemoglobin stabilization, and FACIT-Fatigue score. These findings reflect the results of the global study, in which the non-inferiority of crovalimab was formally verified. Crovalimab also demonstrated a comparable safety profile to eculizumab with no new safety signals in the Chinese subpopulation. Notably, hemolysis control and transfusion avoidance occurred at higher rates in the Chinese subpopulation than in the global population, while BTH occurred at a lower rate. Overall, crovalimab demonstrated its potential as a treatment option for patients with PNH, including Chinese individuals, allowing subcutaneous, self-administered, low-volume administration with maintenance dosing every 4 weeks, improving patient convenience. Moreover, it may be a useful alternative to eculizumab for achieving a lower rate of BTH. Given the exploratory nature of the between-arm comparisons, future studies should formally evaluate whether crovalimab is non-inferior to eculizumab for the treatment of Chinese patients with PNH.

## Supplementary Information

Below is the link to the electronic supplementary material.

Supplementary Material 1

## Data Availability

For up-to-date details on Roche’s Global Policy on the Sharing of Clinical Information and how to request access to related clinical study documents, see: https://www.roche.com/innovation/process/clinical-trials/data-sharing. Anonymized records for individual patients across more than one data source external to Roche cannot, and should not, be linked due to a potential increase in risk of patient re-identification.
