# Supplementary material for: Efficacy and safety of crovalimab versus eculizumab in complement inhibitor-naïve patients with paroxysmal nocturnal hemoglobinuria: Chinese subgroup analysis of the phase 3 COMMODORE 2 study
Source: Ann Hematol. 2026 Jun 13;105(9):385. doi: 10.1007/s00277-026-07129-3 (PMC13490206; doi:10.1007/s00277-026-07129-3)
Supplement: Supplementary file 1 [file 277_2026_7129_MOESM1_ESM.docx]

**Supplementary Methods**

**Main inclusion criteria**

For this Chinese subpopulation analysis, the key inclusion and exclusion criteria were: 1) age ≥18 years at consent; 2) body weight ≥40 kg at screening; 3) paroxysmal nocturnal hemoglobinuria (PNH) diagnosis within 6 months before randomization; 4) complement inhibitor-naïve; 5) lactate dehydrogenase ≥2 × upper limit of normal at screening; 6) presence of ≥1 PNH-related signs or symptoms within 3 months before screening; 7) platelet count ≥30,000/mmat screening without transfusion support within 7 days of laboratory testing; 8) absolute neutrophil count >500/L at screening; and 9) vaccination against Neisseria meningitidis serotypes A, C, W, and Y <3 years before study treatment. Patients were excluded if they had a pre-enrollment hemoglobin concentration ≤7 g/dL, or a pre-enrollment hemoglobin concentration >7 g/dL and ≤9 g/L with concurrent signs and symptoms of anemia.

**Additional safety assessments**

Breakthrough hemolysis, transfusion, and anemia due to PNH were not considered adverse events unless serious; instead, they were recorded as efficacy assessment data. Other safety assessments included changes from baseline in vital signs and clinical laboratory tests, along with the incidence and severity of injection-site reactions, infusion-related reactions, hypersensitivity, and infections.

**Pharmacokinetic and pharmacodynamic assessments**

Serum concentrations of crovalimab and eculizumab over time were measured to evaluate pharmacokinetics. Potential relationships between drug exposure and the efficacy and safety of crovalimab were also assessed.

**Immunogenicity assessments**

The immune response to crovalimab was evaluated by the prevalence of anti-drug antibodies (ADAs) at baseline and the incidence of ADAs. Assessments included the number and proportion of ADA-positive and -negative patients at baseline and after drug administration summarized by treatment, as well as the effect of ADAs on crovalimab exposure.

**Figure S1. Drug administration schedule**

A crovalimab 1000-mg loading dose (patients weighing ≥40 kg to <100 kg) or 1500 mg (patients weighing ≥100 kg) was administered intravenously (Week 1, Day 1), followed by 340 mg subcutaneously (Week 1, Day 2, and Weeks 2, 3, and 4). Subcutaneous maintenance doses of 680 mg (≥40 kg to <100 kg) or 1020 mg (≥100 kg) began at Week 5 and continued every 4 weeks thereafter. Crovalimab self-administration/caregiver administration was permitted from Week 9. If a patient experienced signs and symptoms of PNH, such as breakthrough hemolysis, ≥1 additional intravenous crovalimab doses (recommended 375 mg intravenous, regardless of body weight) was administered.
